# Disseminated *Talaromyces marneffei* infection presenting as multiple intestinal perforations and diffuse hepatic granulomatous inflammation in an infant with STAT3 mutation: a case report

**DOI:** 10.1186/s12879-020-05113-4

**Published:** 2020-06-03

**Authors:** Mianluan Pan, Ye Qiu, Wen Zeng, Shudan Tang, Xuan Wei, Jianquan Zhang

**Affiliations:** 1 Department of Respiratory and Critical Care Medicine, The First Affiliated Hospital of Guangxi Medical University, Nanning, 530021 Guangxi China; 2 Department of Respiratory Medicine, The Eighth Affiliated Hospital, Sun Yat-Sen University, Shenzhen, 518033 Guangdong China

**Keywords:** Gene mutation, Hepatic granulomatous inflammation, Infant, Intestinal perforations, *Talaromyces marneffei*

## Abstract

**Background:**

*Talaromyces marneffei* is a highly pathogenic fungus that can cause life-threatening fatal systemic mycosis. Disseminated *Talaromycosis marneffei* affects multiple organs, including the lungs, skin, and reticuloendothelial system. However, *T. marneffei* infection has rarely been reported in human immunodeficiency virus (HIV)-negative infants with multiple intestinal perforations and diffuse hepatic granulomatous inflammation.

**Case presentation:**

We present the case of an HIV-negative 37-month-old boy who has had recurrent pneumonia since infancy and was infected with disseminated *Talaromycosis.* Contrast-enhanced computed tomography of the whole abdomen showed hepatomegaly and intestinal wall thickening in the ascending colon and cecum with mesenteric lymphadenopathy. Colonoscopy showed a cobblestone pattern with erosion, ulcer, polypoid lesions, and lumen deformation ranging from the colon to the cecum. *T. marneffei* was isolated from the mucous membrane of the colon, liver, and bone marrow. After antifungal treatment and surgery, his clinical symptoms significantly improved. Whole-exome sequencing using the peripheral blood of the patient and his parents’ revealed a heterozygous missense mutation in exon 17 of the STAT3 gene (c.1673G>A, p.G558D).

**Conclusions:**

In *T. marneffei* infection-endemic areas, endoscopic examination, culture, or histopathology from the intestine tissue should be performed in disseminated *Talaromycosis* patients with gastrointestinal symptoms. Timely and systemic antifungal therapy could improve the prognosis. Immunodeficiency typically should be considered in HIV-negative infants with opportunistic infections.

## Background

*Talaromyces marneffei* is a pathogenic fungus that can be disseminated hematogenously to other locations in immunocompromised hosts, especially in adults who are infected with the human immunodeficiency virus (HIV). However, it is increasingly being observed in HIV-negative subjects without obvious risk factors or immunocompromised conditions [1]. The common clinical symptoms associated with *T. marneffei* infections in infants are fever, cough, anemia, lymphadenopathy, hepatosplenomegaly, and being underweight [2]. *Talaromycosis* most commonly involves the lungs, skin, lymph nodes, liver, and spleen [3, 4]. The number of intestinal lymphatics is large, and intestinal can theoretically be the common sites of *T. marneffei* infection. However, *T. marneffei* isolated from tissue biopsies of the colon ulcer has rarely been attributed to intestinal perforation and obstruction. Herein, we report the case of HIV-negative infant who developed disseminated *T. marneffei* infections in the colon, liver, lymph nodes, and bone marrow. His STAT3 gene has heterozygous missense mutations. This case clearly demonstrates etiological evidence of the gastrointestinal manifestations and liver granulomatous inflammation caused by *T. marneffei.*

## Case presentation

**Fig. 1:**
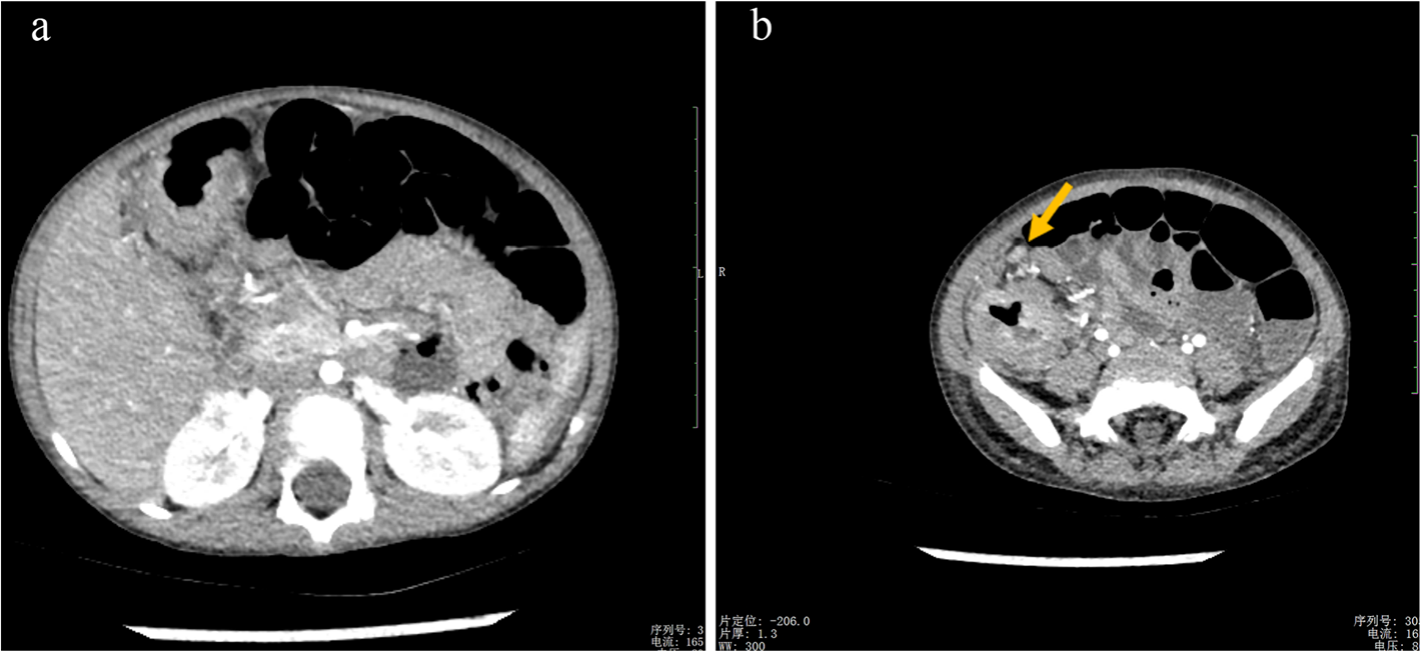
Contrast-enhanced computed tomography of the whole abdomen. **a** Thickening of the intestinal wall in the ascending colon, and (**b**) mesenteric lymphadenopathy in the ileocecal region can be seen. The arrowhead indicates the mesenteric lymphadenopathy

**Fig. 2:**
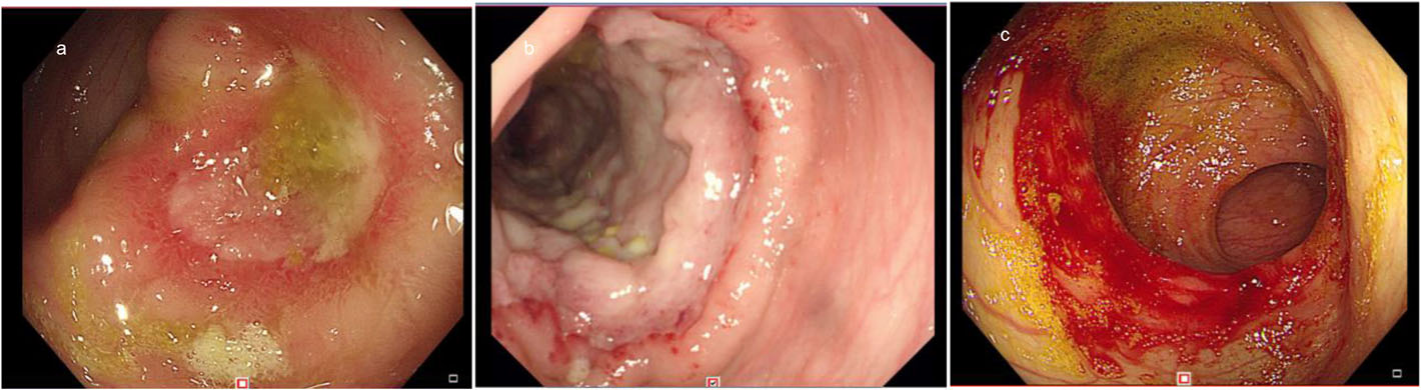
**a** Colonoscopy showing a huge ulcer in the cecum and flushed and swollen surrounding mucosa, and **b** polypoid lesions. **c** Repeat colonoscopy after treatment shows good recovery of the stoma, located at the ileocecal region, within 35 cm from the anal verge

**Fig. 3:**
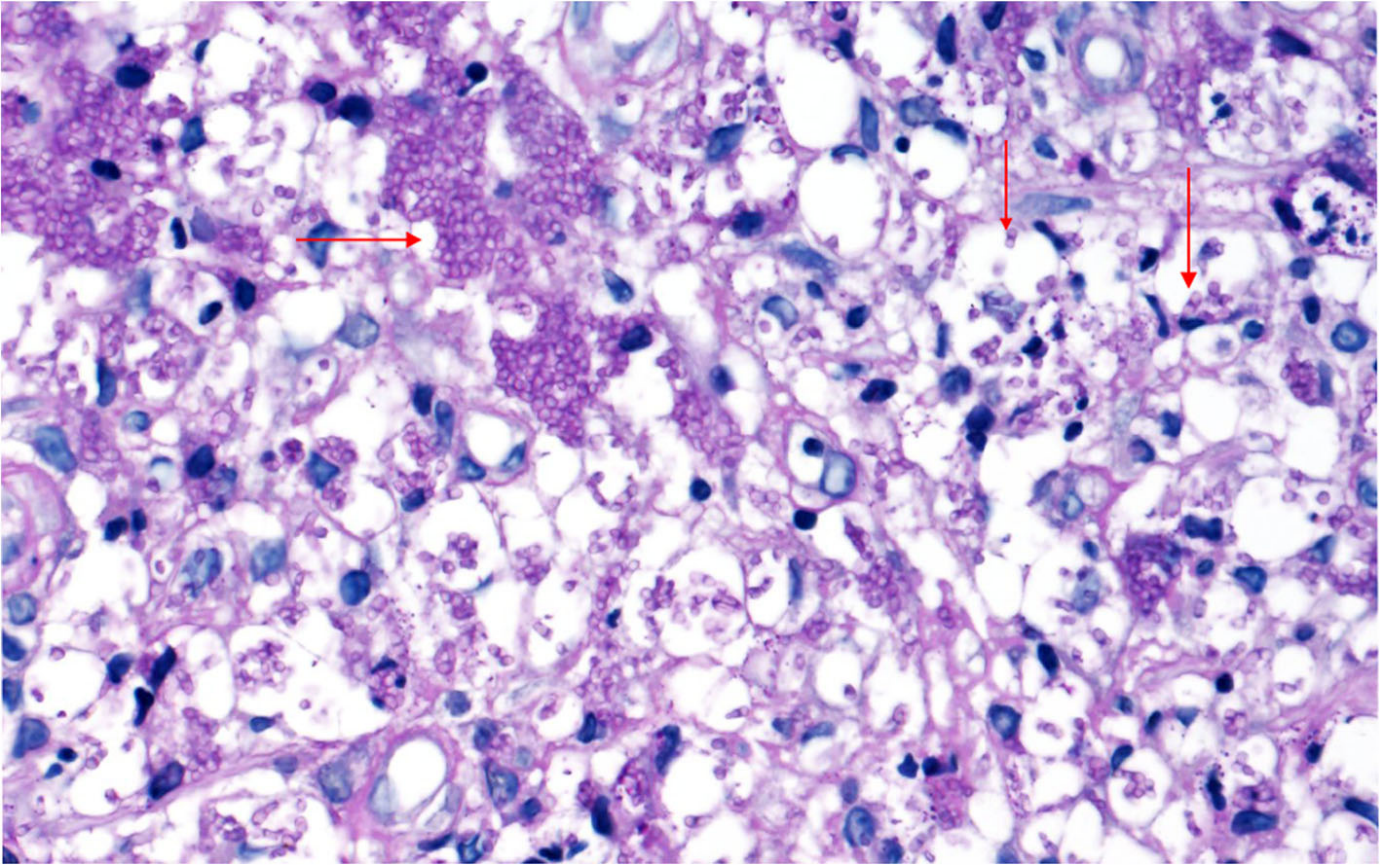
The yeast form of *T. marneffei* was confirmed by the histopathological analysis of the intestine using periodic acid-Schiff (PAS) staining. (magnification × 400)

**Fig. 4:**
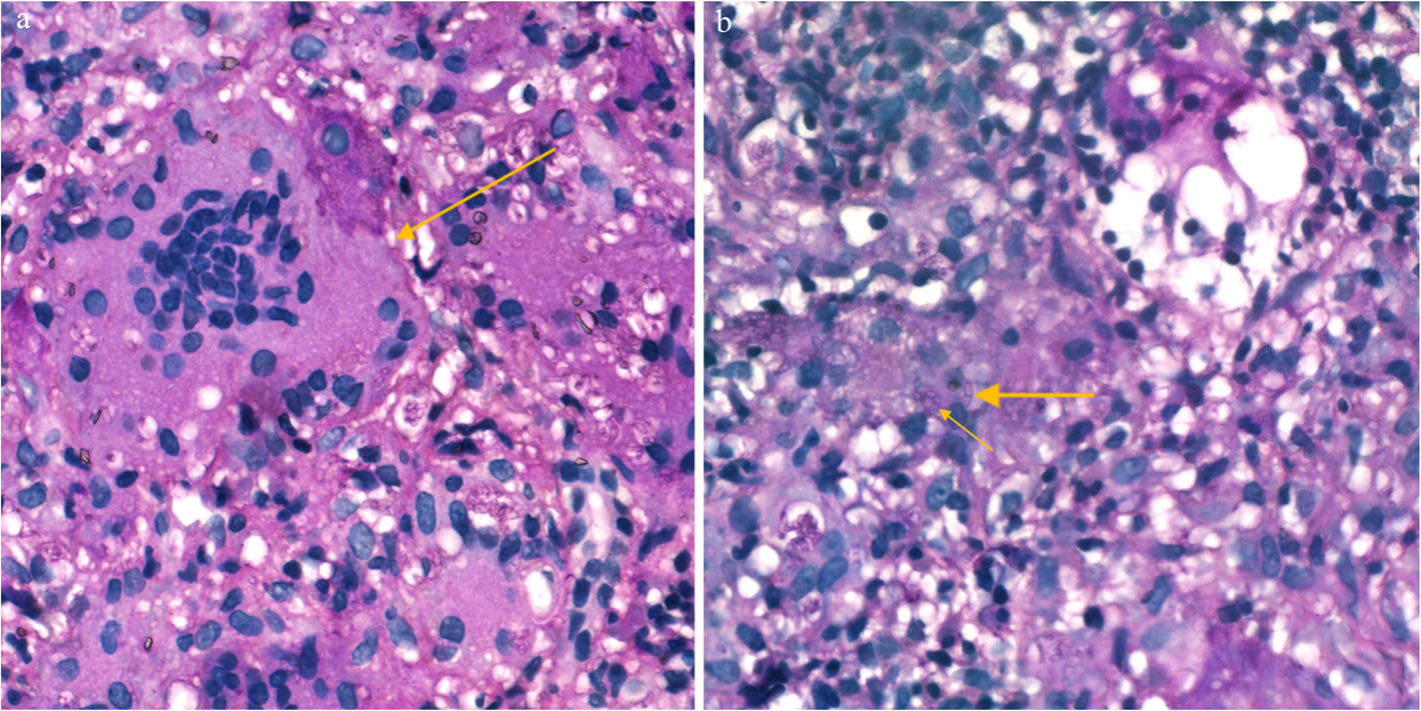
**a** Granulomatous inflammation observed during histopathological examination of the liver. **b** PAS staining of liver tissue revealed numerous intracellular yeast-like or sausage-like cells 2–4 μm in diameter with a transverse septum (arrows) (magnification × 400)

**Fig. 5:**
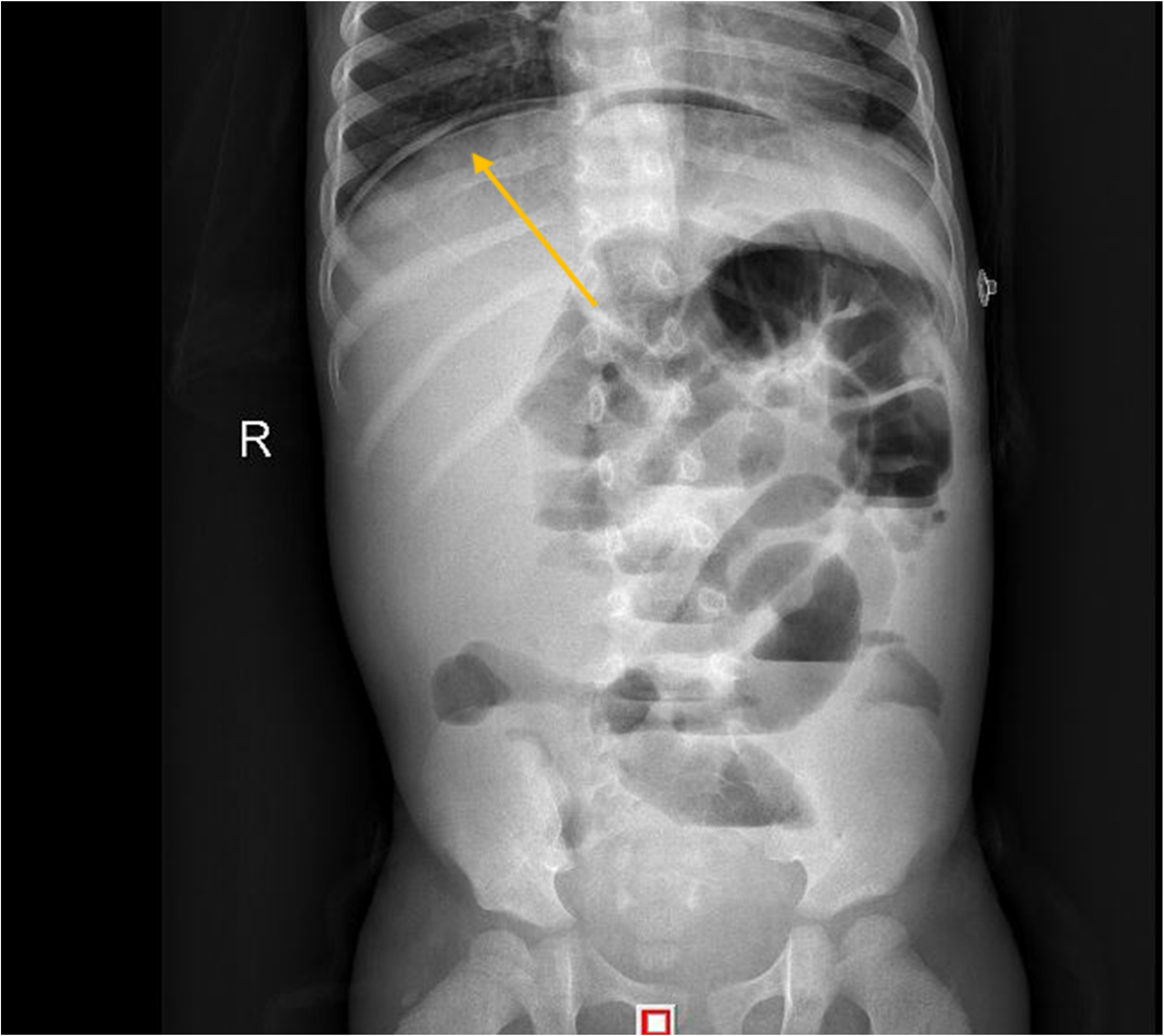
Abdominal radiograph showing arc-shaped gas density shadows, intestinal inflatability, intestinal dilatation, and multiple gas-liquid planes under the diaphragm

**Fig. 6:**
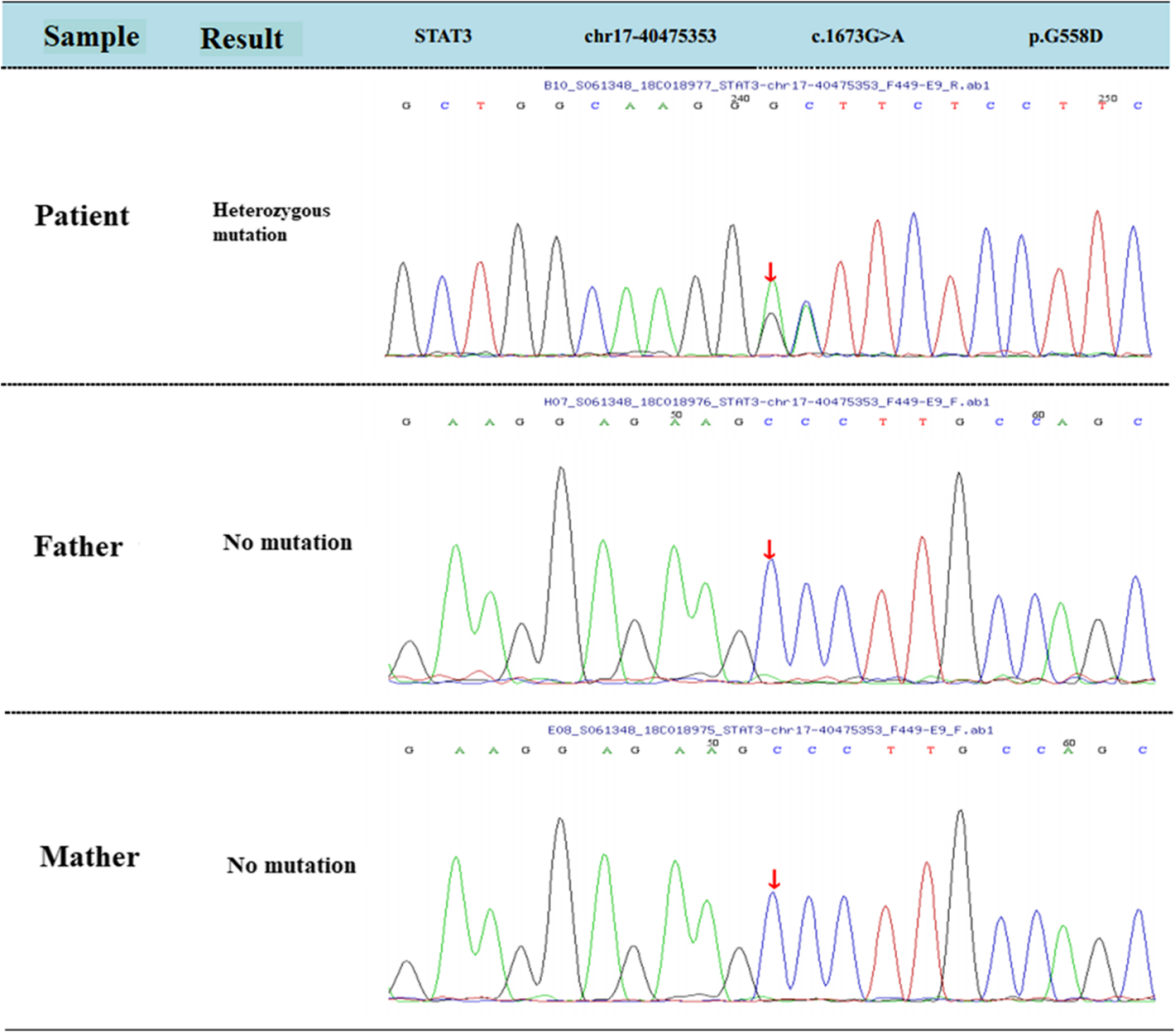
The mutations found in patients with *T. marneffei* infections. Whole-exome sequencing indicate a STAT3 missense mutation of C to G

A 37-month-old male infant from Guangxi Province, China, with recurrent pneumonia since infancy exhibited recurrent pain in the right abdomen and fevers for 3 months. The abdominal pain was related to eating food, but not to physical activity and position. His maximum temperature was 40 °C. Physical examination revealed lymphadenopathy in the left neck and hepatomegaly. His abdomen was soft with normal bowel sounds; however, the liver and spleen were palpable 4 cm below his costal margins. The rest of the physical examination was unremarkable. Routine blood tests revealed 16.9 × 10^9^/L leucocytes, 9.5 × 10^9^/L neutrophils, 4.4 × 10^9^/L lymphocytes, 0.22 × 10^9^/L eosinophils, and 90 g/L hemoglobin. Blood was observed in his stool. The serum albumin and C-reactive protein levels were 29.0 g/L (40–60 g/L) and > 192 mg/L (< 10 mg/L), respectively. Erythrocyte sedimentation rate was 28 mm/h (≤15 mm/h). Serum aspergillus galactomannan antigen was 0.826 (normal<0.5). His CD4+ T-cell count was 1078 cells/μL (normal: 410–1590 cells/μL), while the percentage of his natural killer cells was 18.10% (normal: 9–15%). Serum immunoglobulin (Ig) M was slightly elevated, while IgG and IgA were normal. Serum aspartate aminotransferase, alanine aminotransferase, and creatinine levels, as indicated by the blood test, were all normal. Anti-nuclear and anti-HIV antibodies and INF-γ autoantibody were all negative. Blood and stool cultures were negative. Chest computed tomography (CT) showed disseminated patchy exudates throughout the left upper and lower lobes of the right lung. Contrast-enhanced CT of the whole abdomen showed hepatomegaly, intestinal wall thickening in the ascending colon, and mesenteric lymphadenopathy in the cecum with (Fig. 1a-b). Colonoscopy showed a cobblestone pattern (non-ulcerated mucosa separated by ulcers) with erosion, ulcer, polypoid lesions, and lumen deformation from the colon to the cecum (Fig. 2a-b). Crohn^’^s disease and intestinal tuberculosis were also considered based on the presence of multiple ulcers on colonoscopy and on the clinical manifestations. Therefore, the patient was treated with mesalazine and thalidomide for 1 week but without clinical improvement. After 5 days, the colon biopsy revealed mucosal ulceration and ulcers in the colon and massive infiltration of the mucosa and submucosa by the engorged macrophages. A large number of fungal spores were observed in the interstitial space and the macrophages (Fig. 3). Subsequently, the patient underwent ultrasound-guided liver biopsy. Histopathological examination of the liver tissue revealed granulomatous inflammation (Fig. 4a), while periodic acid-Schiff staining revealed aggregates of macrophages engorged with numerous yeast-like organisms 2–4 μm in diameter. These yeast-like organisms were spherical to oval and had a transverse septum (Fig. 4b). After 2 weeks, the bone marrow culture confirmed *T. marneffei,* and the diagnosis of disseminated *Talaromycosis* involving the liver, colon, lymph nodes, and bone marrow was made. Intravenous voriconazole (12 mg/kg every 12 h) was administered for 4 weeks. Subsequently, the liver size reduced and was palpable 1 cm below the costal margin. Voriconazole was then administered orally (7 mg/kg twice a day). Unfortunately, 1 month later, his abdominal pain and fever recurred, and he presented with reduced urine output. Abdominal radiograph showed bowel perforation, pneumoperitoneum, and intestinal obstruction (Fig. 5). Hence, an emergency exploratory laparotomy with intestinal resection, anastomosis, and a colostomy was performed. During the surgery, we observed the pebble sign with erosion in the ileocecal intestinal cavity. The lesion segment was approximately 8 cm long. The ileocolic junction was narrow and obstructed, and the adjacent intestinal ducts were edematous and thickened. Postoperative pathology indicated the presence of *T*. *marneffei.* Whole-exome sequencing was performed using the patient’s and his parents’ peripheral blood. A heterozygous missense mutation in exon 17 of the STAT3 gene (c.1673G>A, p.G558D) was found in the patient but not in his parents (Fig. 6), indicating that the mutations were de novo.

The patient’s condition improved after the administration of intravenous voriconazole and antibiotics for 10 days and oral voriconazole for 7 months. Seven months after the surgery, repeat colonoscopy showed good recovery of the stoma located at the ileocecum, within 35 cm from the anal verge (Fig. 2c). No relapse was observed during the 18-month period of antifungal treatment.

## Discussion and conclusions

Here we report the clinical course, diagnosis, and management of an HIV-negative infant who was infected with *T*. *marneffei.*

*T*. *marneffei* is a dimorphic fungus that causes disseminated infection in endemic regions. In mainland China, the Guangxi Province is an endemic area for *T*. *marneffei* infections [5]. Primary *Talaromycosis* has been commonly reported in adult patients with acquired immune deficiency syndrome, but HIV-negative infants infected by *T*. *marneffei* have rarely been described in the literature. Disseminated infection of *T*. *marneffei* usually affects the reticuloendothelial system of multiple organs, including the skin, lungs, bones, bone marrow, lymph nodes, liver, and spleen. Infections in the mesenteric and central nervous systems and the main trachea have also been reported [4, 5]. However, infants with STAT3 mutations who developed disseminated *Talaromycosis* involving the colon experienced serious perforated viscus complications, but hepatic granulomatous inflammation has never been reported.

Gastrointestinal *Talaromycosis* clinical presentations include abdominal pains, diarrhea, or bloody stool. Colonoscopy on the present patient showed a cobblestone pattern with erosion, ulcer, polypoid lesions, and lumen deformation; however, stool culture was negative. The initial clinical presentation was not indicative of *Talaromycosis*, although he resided in a *T*. *marneffei* infection-endemic area. Instead, Crohn^’^s disease, intestinal tuberculosis, and other non-infectious etiologies, including lymphoma, were suspected. Ultimately, *T*. *marneffei* was isolated from the colectomy specimen. *T*. *marneffei* usually invades the mononuclear macrophage system. However, reports on *T*. *marneffei* infection involving the intestine are scarce, and physicians may overlook *T*. *marneffei* invasion of the intestinal tract, which may lead to intestinal perforation or other serious complications. Therefore, we must be vigilant of the possibility of *T. marneffei* infections involving the intestine in patients with gastrointestinal manifestations who live in endemic areas. Even if the stool culture is negative, abdominal CT or endoscopic examination should be performed to confirm intestinal *T*. *marneffei* invasion. Timely culture and histopathological examination of specimens from the mucous membrane of the colon are crucial for diagnosis and differential diagnosis.

In this case, histopathological examination of the liver presented with granulomatous inflammation indicating the presence of yeast in the cytoplasm of the giant cells. Hepatic lesions of *T*. *marneffei* infection showed three pathological patterns: diffuse, granulomatous, and mixed [6]. The granulomatous pattern is often seen in the organs of the reticuloendothelial system of patients with normal immunity. Animal models have shown that immunocompetent mice developed a granulomatous response to the organism with its eventual elimination. However, congenitally immunodeficient mice initially formed granulomata with the replacement of the liver parenchyma by free yeast-like cells and proliferation of macrophages containing yeast-like organisms, which eventually disseminated to other locations [7]. In our study, the granuloma formation pattern was seen in the patient. *T*. *marneffei* multiplies in the liver macrophages, which proliferate and enlarge to accommodate the proliferating fungi [6].

Bamboo rats are natural hosts of *T. marneffei*. Our patient’s parents were unaware of any rodent exposure; hence, the route by which he acquired the *T. marneffei* infection was uncertain. Previous studies have shown that *T*. *marneffei* infection in HIV-negative children may be related to immunodeficiencies, such as Th17 deficiency, autosomal dominant gain-of-phosphorylation signal transducer and activator of transcription STAT1 mutations, and STAT3 heterozygous missense mutations [1, 8]. The patient in our study has a STAT3 deficiency and is the third reported case of *Talaromycosis* [1, 9], indicating that gene mutation may increase the risk of fungal infection. Literature on the genetic susceptibility associated with *T*. *marneffei* infection is lacking and is worth studying in greater detail.

Amphotericin B, itraconazole, and voriconazole are effective in treating *Talaromycosis* [10]. The recommended dosage and duration of treatment for HIV-infected adult patients are intravenous amphotericin B deoxycholate 0.6–1.0 mg/kg/day for 2 weeks, followed by oral itraconazole 400 mg/day for 8–10 weeks [11]. However, for HIV-negative children, there is still no unified standard therapy. The adverse effects of amphotericin B, especially nephrotoxicity and hepatotoxicity, occur frequently and limit its clinical application in children [12]. A study in HIV-negative children in Southern China showed that voriconazole has high levels of efficacy and is a well-tolerated therapeutic option for disseminated *T. marneffei* infection in HIV-negative children [13]. We also have reported that 7 HIV-negative children achieved voriconazole with good results [2]. Initially, the patient in this study received voriconazole antifungal therapy and showed significant improvement in symptoms. However, even during treatment, abdominal pain and fever recurred and progressed to yield severe perforated viscus complications. The clinical outcome is closely related to the patient’s basic status, immune function status, and organ function. The patient’s abdominal CT showed intestinal wall thickening, and colonoscopy showed multiple ulcers. It was not clear at that time whether *T*. *marneffei* infection had invaded the entire intestinal wall, but it ultimately progressed to perforation. We suggest performing a colonoscopy to find ulcers and recommend performing intestinal magnetic resonance examination. If *T*. *marneffei* invades the intestinal muscle, surgery to remove the gut lesion can also reduce the fungal burden that may be done to avoid intestinal perforation. Finally, after 7 months of regular antifungal therapy and surgery, the symptoms, signs, and colonoscopy findings showed significant improvements. Antifungal therapy should be administered early on, with broad-spectrum coverage, and for a prolonged period to control the infection in patients with *Talaromycosis*. Moreover, note that it is vital to perform the appropriate surgery in a timely fashion.

This is the first reported case of disseminated *T*. *marneffei* infection in an infant with STAT3 gene deficiency, leading to multiple ulcers in the colon, intestinal perforation, and diffuse hepatic granulomatous inflammation. Clinicians should pay more attention to disseminated *Talaromycosis* with gastrointestinal symptoms in *T. marneffei* endemic areas and should perform endoscopic examination and culture or histopathology. Timely and systemic antifungal therapy could improve the prognosis. The mutation in the STAT3 gene suggests that the patient might have genetic susceptibility to fungal infections.

## Data Availability

All data generated or analyzed during this study are included in this published article.

## Abbreviations

HIV: Human immunodeficiency virus
Ig: Immunoglobulin
CT: Computed tomography
